# Cochlear Aqueduct Advection and Diffusion Inferred from Computed Tomography Imaging with a Bayesian Approach

**DOI:** 10.1007/s10162-026-01047-x

**Published:** 2026-04-13

**Authors:** Amal M. A. Alghamdi, Barbara K. Mathiesen, Leo Miyakoshi, Maiken Nedergaard, Jakob S. Jørgensen, Peter A. R. Bork

**Affiliations:** 1 Department of Applied Mathematics and Computer Science, Technical University of Denmark, Richard Petersens Plads, Building 324, Kongens Lyngby, 2800 Denmark; 2 Center for Translational Neuromedicine, University of Copenhagen, Blegdamsvej 3B, Copenhagen, 2200 Denmark; 3 Department of Physics, Technical University of Denmark, Building 307, Kongens Lyngby, 2800 Denmark

**Keywords:** Cerebrospinal fluid, Advection–diffusion, Cochlear aqueduct, Bayesian parameter inference

## Abstract

**Purpose:**

Fluid flow and transport of therapeutic agents and pathogenic bacteria into the cochlea has been challenging to study due to its small size and location within bone. We here take advantage of recent non-invasive Computed Tomography (CT) imaging to infer transport parameters in a 1-dimensional advection–diffusion model of the cochlear aqueduct using a Bayesian approach.

**Methods:**

Six male C56BL/6 mice injected with the small molecule tracer iohexol in cisterna magna were scanned every 5 min for 30 min in CT under anesthesia. Using the CT data to set boundary conditions, we solve the advection–diffusion equation for given advection and spatially varying diffusion parameters. The CT data is modeled as normally distributed around the model-predicted concentration. We specify priors for the model unknown parameters and infer their posterior distributions using Bayes’ formula and the CUQIpy library. The statistical approach is validated using synthetic data.

**Results:**

The evolution of the concentration of tracer in the cochlear aqueduct is well predicted by the advection–diffusion model using inferred parameters. Though diffusion of the small CT tracer varies along the aqueduct, it is usually near the free diffusion and therefore not likely to be influenced much by membranes or flow. Advection is inferred near zero in most cases. We show how to use these results to calculate transport through the cochlear aqueduct for other animals and molecules.

**Conclusion:**

Free diffusion dominates transport of small molecules in the cochlear aqueduct, which can therefore be effectively approximated using simple 1-dimensional (advection-)diffusion formulas.

**Supplementary Information:**

The online version contains supplementary material available at 10.1007/s10162-026-01047-x.

## Introduction

The cochlear aqueduct is a small channel which connects the perilymph fluid of the cochlea with the cerebrospinal fluid of the subarachnoid space surrounding the brain. The aqueduct may ossify during adulthood, but is mostly open in children [1–3]. The aqueduct has recently been used as a delivery route of viral gene therapy that rescues hearing in mice and primates [4, 5]. Ongoing clinical trials in humans use direct injection into the cochlea, but this delivery route risks local damage, and the aqueduct may be preferable [4, 6, 7]. Transport of bacteria through the aqueduct may explain that about 10% of bacterial meningitis cases develop hearing loss, and viral meningitis is also associated with hearing loss  [8–10]. Such transport has been shown to be inhibited by a mesh of arachnoid-like tissue, which partly occupies the aqueduct [11] corresponding to the recently identified subarachnoid lymphatic-like membrane [4, 12, 13]. Due to the small size and location within the bone, the mechanisms of transport through the cochlear aqueduct of fluid, solutes, and large particles remain unclear.

**Fig. 1:**
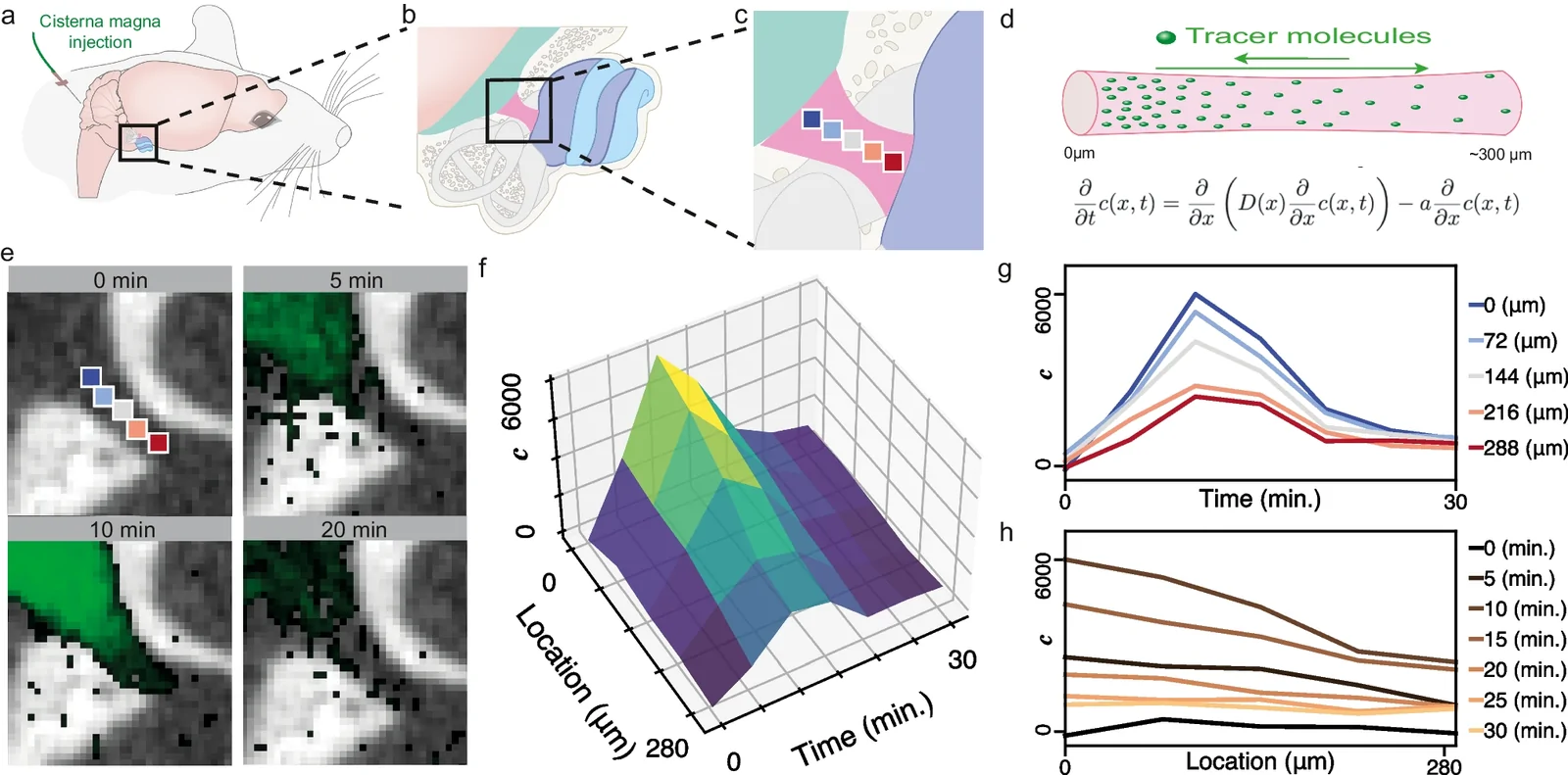
Computed Tomography imaging of the evolution of a small tracer in the mouse cochlear aqueduct allows modeling of transport modes. **a** Sketch of the inner ear within the mouse head and cisterna magna injections. **b** Zoom-in showing the cochlear aqueduct (pink) connecting the cochlea (dark and light blue) and the sub-arachnoid space (green). **c** Zoom-in of the cochlear aqueduct with markings of the regions of interests (ROIs) where data concentration measurements are collected. **d** Schematic representation of the tracer transport model in the cochlear aqueduct. **e** CT images of the tracer (green) moving through the cochlear aqueduct acquired at 5 min increments with markings of the ROIs. **f** 3D representation of tracer concentration over space (the cochlear aqueduct) and time (30 min window). **g** 2D representation of the data shown in **f** showing concentration over time for different locations. **h** 2D representation of the data shown in **f** showing concentration over space for different times. **c** Concentration, arbitrary unit. **a**–**d** Reprinted with alterations from [4] with permission (license number 6051950723672)

Using exsanguination and sampling of perilymph via puncturing of the cochlea, Kellerhals estimated in the late ’70 s that cerebrospinal fluid inflow via the cochlear aqueduct contributes about a fifth of perilymph production in guinea pigs, with the remaining four-fifths entering and leaving by the blood [14, 15]. More recently, several studies using injection of tracers into the cochlea along with sampling at various locations have estimated flow rates ranging from 0.05nl/min to 30nl/min in the aqueduct from the subarachnoid space to the cochlea of guinea pigs [16–18]. To tease out physiological flow from the effects of invasive injections and samplings, elaborate compartment models with tens of parameters have been fitted to data [18]. But fluid flow and solute transport through a narrow intact aqueduct are completely determined by only channel dimensions, fluid pressure, and diffusion, raising the opportunity for simpler models of non-invasive tracer transport.

We here use computed tomography (CT) imaging recently published [4] to take advantage of the simpler physics of transport in the intact cochlear aqueduct (Fig. 1a–d). The data was acquired in mice by injection of a small (0.8kDa) CT contrast agent iohexol into the cisterna magna and subsequent CT imaging of the cochlear aqueduct under ketamine-xylazine anesthesia [4]. Tracer signal arrived within a few minutes at the cochlear aqueduct, then filled the aqueduct and entering the scala tympani at about 10 min post-injection before finally fading out (see Fig. 1e–h).

Tracer signal measurements contain noise inherent to the imaging technology and movement of the animal in the scanner, leading to uncertainty in the determination of transport parameters. While most studies of cochlear aqueduct transport deal with noise by averaging over several animal experiments, that approach removes information and risks finding zero flow on average when animals show forward and backward flows. Uncertainty quantification has been used to compare magnetic resonance tracer transport in humans with a diffusion-based model, but parameter inference is too computationally demanding for such large models [19, 20]. For the smaller mouse brain, one study has previously employed both uncertainty quantification and Bayesian parameter inference, but that model was limited to constant diffusion [21].

We here use Bayesian parameter inference with uncertainty quantification for advection with spatially varying diffusion in recordings of individual mouse cochlear aqueducts in order to determine how molecules are transported through the cochlear aqueduct. In the “Transport Model and Bayesian Inference of Spatially Varying Advection and Diffusion” section, we present the aqueduct transport model and statistical inference approach. In the “Parameter Inference and Data Prediction Can Detect Slow Advection with Spatially Varying Diffusion in Synthetic Data” section, we show that the statistical approach can identify true parameters in simulated transport data, in the “The Model Successfully Predicts CT Data with Inferred Transport Parameters” section that the CT data can be successfully predicted with the model, in the “Inferred Transport Parameters Indicate that Diffusion Dominates for Small Tracers” section that diffusion dominates transport for small molecules, and in the “Approximate Closed-form Solution for Cochlear Aqueduct Transport of Larger Particles with Constant Diffusion” section, we give a formula with which researchers can calculate the rate of transport through the cochlear aqueduct for given molecular and aqueduct sizes. The “Discussion” section discusses the results.

## Results

### Transport Model and Bayesian Inference of Spatially Varying Advection and Diffusion

The evolution of the concentration of a small inert solute such as the CT tracer in a fluid is, in general, governed by diffusion due to thermal molecular motion and advection due to the flow of the fluid solvent. Diffusion in free water at slow speeds can be described with a single diffusion coefficient, which Einstein showed in 1905 to be proportional to temperature *T* and inversely proportional to molecular radius *r* and fluid viscosity η [22],

1 $$\begin{aligned} D_E = \frac{k_B T}{6 \pi \eta r}, \end{aligned}$$

where $k_B$ is the Boltzmann constant (roughly $1.4\times 10^{-23}$J K$^{-1}$). With an approximate r≈0.89nm for iohexol, $D_E \approx 368$µm$^2$/s, which agrees with an experimental report [23] (using $\eta \approx 7 \times 10^{-4}$Pa s [24] and T=310K, with the experiment lower than in free water due to its agar medium). Adjustments for extracellular interstitial spaces in the brain have been developed more recently, which use factors for fluid volume fraction and tortuosity to modify the formula for the free diffusion coefficient [25, 26]. Fluid volume fraction and tortuosity may change along the cochlear aqueduct due to meshes, anatomical structure, or ossification, leading to a spatially varying diffusion coefficient. Simple well-developed flows in elongated tubes like the cochlear aqueduct will have parabolic velocity profiles, which can increase the macroscopic appearance of diffusion in what is known as Taylor-dispersion, which again modifies the diffusion parameter [27]. But oscillations such as those reported previously for flow in the cochlear aqueduct [18, 28] can make the velocity profile flatter as the frequency of oscillation increases [29]. The macroscopically observable rate of diffusion is called the effective diffusion. With these considerations in mind (see methods “Modeling the Bayesian Inverse Problem” section), we model the evolution of cochlear aqueduct tracer concentrations *c*(*x*, *t*) over time *t* with a one-dimensional advection–diffusion equation in which the effective diffusion parameter *D*(*x*) may vary along the aqueduct position *x* (see Fig. 1d).

2 $$\begin{aligned} \frac{\partial }{\partial t}c(x,t) = \frac{\partial }{\partial x}\left( D(x) \frac{\partial }{\partial x} c(x,t) \right) - a \frac{\partial }{\partial x}c(x,t). \end{aligned}$$

Here, *a* is the (time-average) velocity of advection, which is constant in space to conserve fluid mass.

Since particle size influences diffusion, it is convenient to evaluate the relative contribution of advection compared to diffusion using the Péclet number $\textrm{Pe}$:

3 $$\begin{aligned} \textrm{Pe} = \frac{a x^\text {max}}{\bar{D}}, \end{aligned}$$

Here, $x^\text {max}$ is the length of the cochlear aqueduct, and $\bar{D}$ is the average effective diffusion coefficient. A Péclet number with magnitude less than one implies that diffusion contributes more to net transport for the particular solute, and a negative value indicates flow from cochlea back towards subarachnoid space. The observed effective diffusion parameter may contain contributions from dispersion, microscopic and oscillating velocity profiles, and tissue obstruction.

In the case of the CT data recently produced to investigate transport in the cochlear aqueduct [4], only a signal roughly proportional to tracer concentration is directly available, whereas the transport parameters must be inferred from their effect on the concentration. We developed a Bayesian approach to inferring the transport parameters while taking CT imaging noise into account (see methods “Modeling the Bayesian Inverse Problem” section). Briefly, we took CT signal measurements to be governed by the advection–diffusion Eq. 2 with additive Gaussian noise, and placed priors on the noise level, velocity, and varying-in-space diffusion parameters. We then applied Bayes’ formula and numerically estimated the data-informed posterior probabilities of the parameters using the CUQIpy library [30, 31]. The complete data and code in the form of Jupyter notebooks for reproduction of our results are open-source and available online [32]. It is additionally useful for the more general problem of inferring one-dimensional advection–diffusion parameters.

**Fig. 2:**
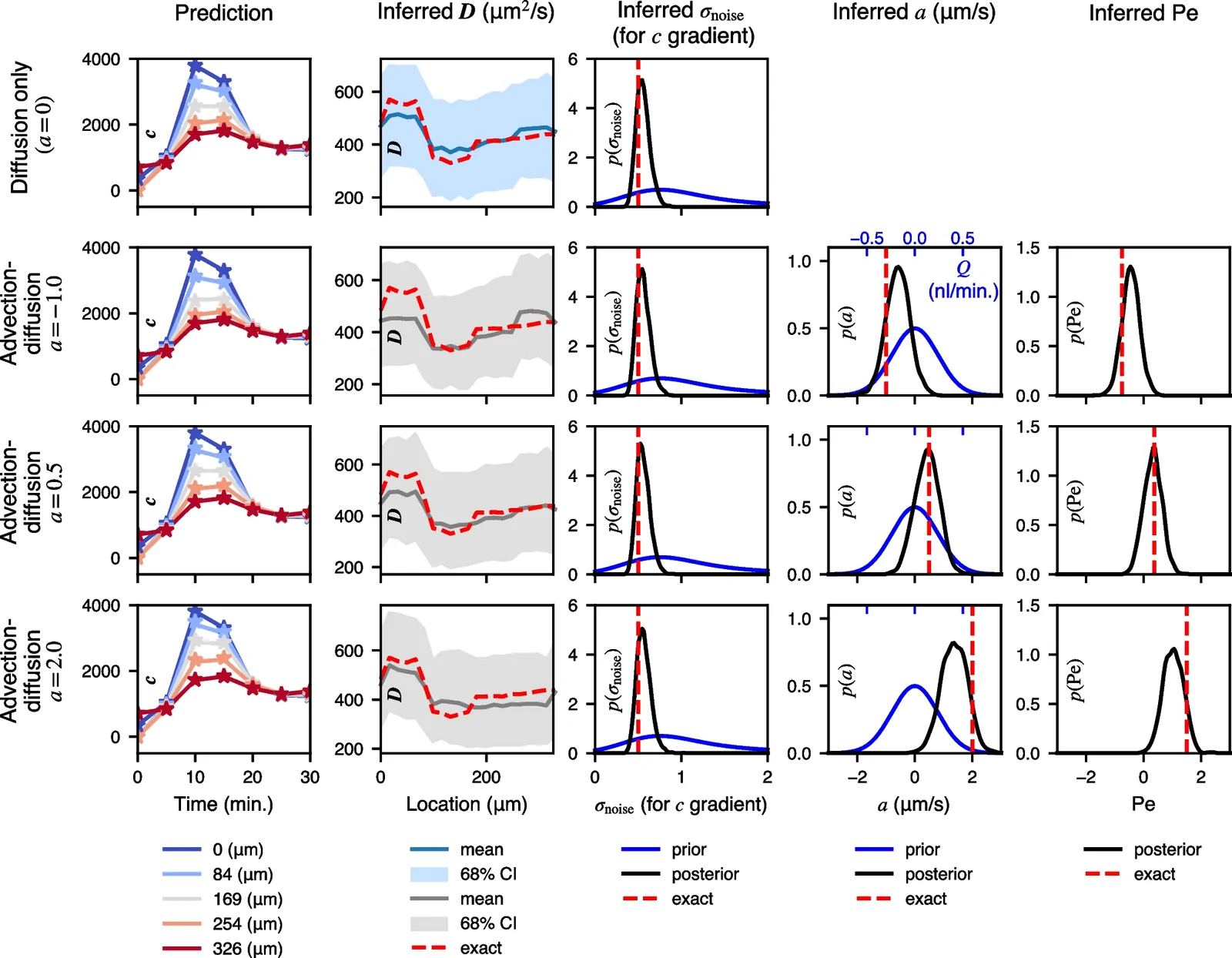
The Bayesian inference setup correctly identifies spatially varying diffusion, advection, and noise in synthetic data for a diffusivity profile of relatively low diffusivity in the middle of the aqueduct. Each row represents a different setup of the inference problem: diffusion only (first row), advection of -1 µm/s. (second row), advection of 0.5 µm/s. (third row), and advection of 2 µm/s. (fourth row). The first column shows the predicted data (solid lines) and the exact data (marked as stars). The second column shows the 68% credibility interval plot of the inferred varying in space diffusivity (blue for the diffusion-only case and gray for the advection–diffusion cases), the exact diffusivity profile (red), and the posterior-mean diffusivity profile (blue for the diffusion-only case and gray for the advection–diffusion cases). The third column shows the prior (blue) and posterior (black) and exact value (red dashed line) of the noise standard deviation. The fourth column shows the prior (blue) and posterior (black) and exact value (red dashed line) of the scalar advection speed along with volume flow rate *Q* (based on measured cochlear aqueduct cross-section). The fifth column shows the posterior (black) and exact value (red dashed line) of the Peclet number, Eq. 3. The minimum component-wise effective sample size for all inferences is ∼1000

### Parameter Inference and Data Prediction Can Detect Slow Advection with Spatially Varying Diffusion in Synthetic Data

To validate the modeling approach and its ability to detect slow advection and spatially varying diffusion, we generated synthetic data using model Eq. 2 and inferred the parameters retroactively, see Fig. 2 (and methods). Though the concentration profiles corresponding to advective velocities *a* of 0, -1µm/s, 0.5µm/s, and 1µm/s are visually difficult to distinguish (column 1), our Bayesian approach correctly infers the noise, diffusion, and advection parameters (columns 2–4). Even for slow advection corresponding to Péclet number less than one, the velocity is correctly inferred (columns 4 and 5). In supplementary material (included in appendix for reviewing purposes, see Figs. A1–A2), we show similar results for other choices of true spatial variation in the diffusion coefficient. This shows that in so far as the advection–diffusion equation holds, our Bayesian inference methods are able to detect both slow advective and spatially varying diffusive contributions to cochlear aqueduct transport.

**Fig. 3:**
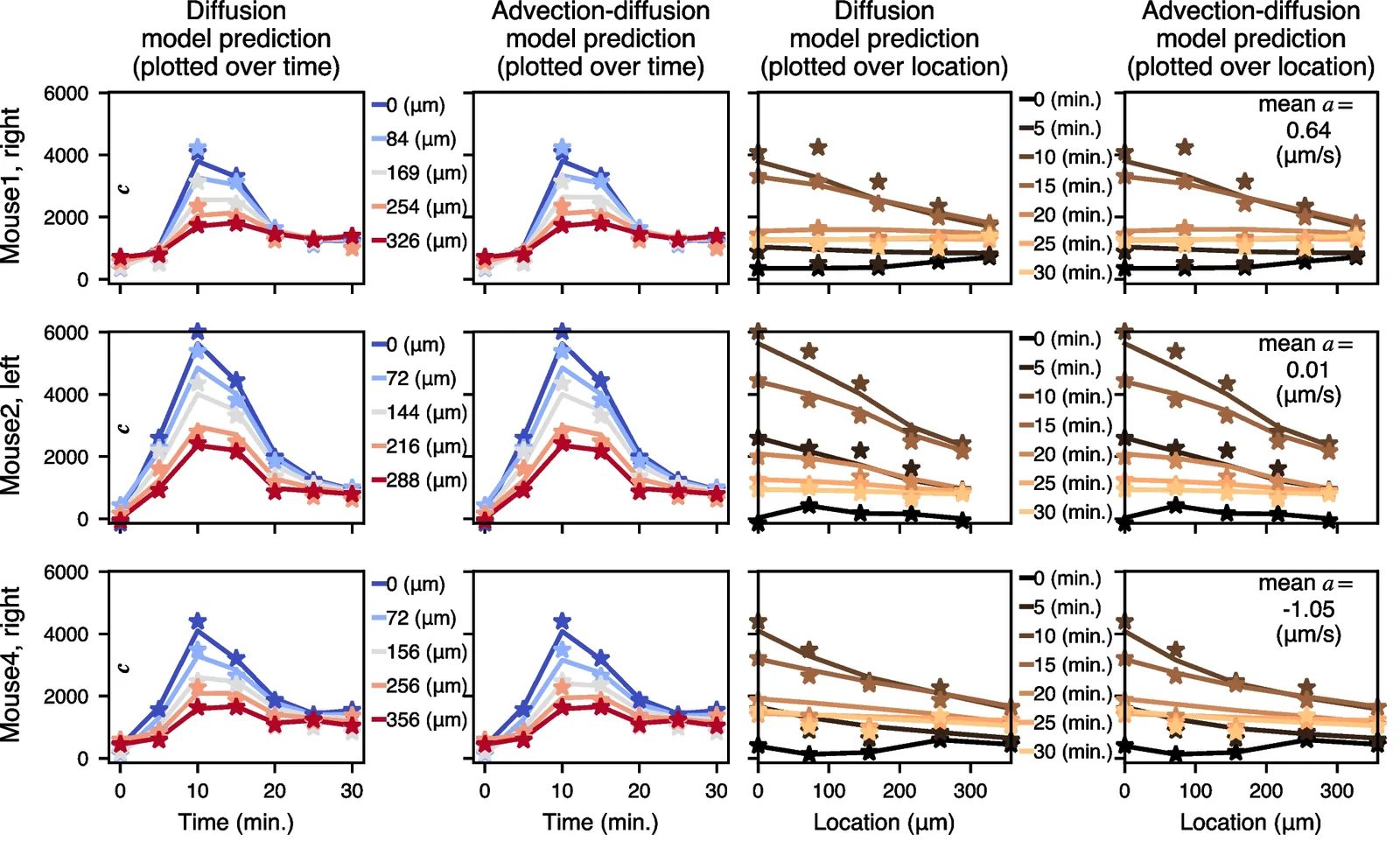
The inferred evolution of tracer concentration in cochlear aqueduct based on real data with and without advection assumption aligns well with the data. Each row shows a prediction for a particular real-data case as labeled. For example, the first row corresponds to animal 1 right ear. The first column shows our diffusion-only model prediction, while the second column shows our advection–diffusion model prediction. Prediction is shown as solid lines, and the CT data are marked by stars. The third and fourth columns show the same data in the first and second columns, respectively, but with different visualization: plotting concentrations at specific times over location instead of plotting concentrations at specific locations over time

### The Model Successfully Predicts CT Data with Inferred Transport Parameters

We next applied our inference method to CT data of tracer evolution in the cochlear aqueduct of anesthetized mice. With the inferred parameters, the model correctly predicts the data, see Fig. 3 for representative cases selected to illustrate positive, negative, and no inferred advection (and Fig. A3 for remaining cases). In columns 1 and 2, the predicted evolution of concentration in solid lines is overlaid with matching stars representing data (colored by location). Advection contributes slightly in the top and bottom rows, but the predictions are very similar to the diffusion-only predictions. Prediction errors are small, and the model captures the evolution of concentration in the cochlear aqueduct (see Fig. A4).

With diffusive transport, the local rate of change is proportional to the spatial curvature on the concentration profile (see Eq. 2). Figure 3 and A3 columns 3 and 4 therefore show concentration profiles over location. In contrast to diffusion, advective transport rather translates the spatial concentration profile along the first axis. Such translation of the concentration profile in space is not seen in our recordings, even in the cases with the most rapid advection (fourth column, third row). Rather than being translated along the axis, steep spatial gradients correspond to locations with a rapid rate of change in concentration (see, e.g., locations near 0–100µm at times 0–10 min where both the spatial and temporal slopes are steep). The data and model, therefore, qualitatively reflect that diffusion dominates the transport of the small CT tracer.

**Fig. 4:**
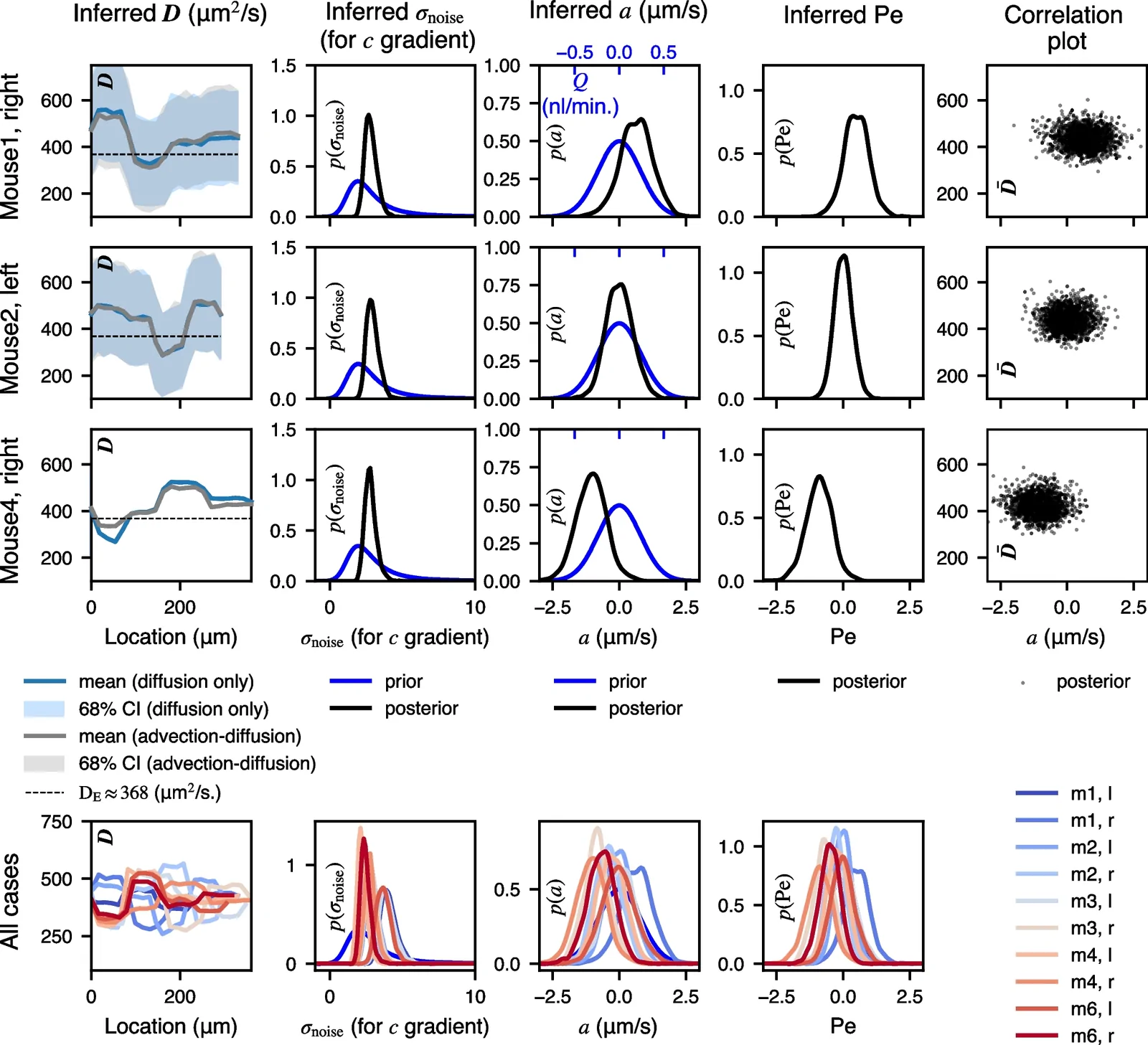
Bayesian parameter inference of spatially varying diffusion near the free diffusivity and small advection levels. The first three rows show inference of three cases, respectively, as labeled. In each of those rows, the first column shows the 68% credibility interval plot of the inferred varying in space diffusivity (blue for the diffusion-only inference case and gray for the advection–diffusion inference case), the posterior-mean diffusivity profile (blue for the diffusion-only case and gray for the advection–diffusion case) along with $D_\text {E}$ value (gray dashed line). The second column shows the prior (blue) and posterior (black) of the inferred noise standard deviation $\sigma _\text {noise}$. For simplicity, we only show $\sigma _\text {noise}$ posterior for the advection–diffusion case. The third column shows the prior (blue) and posterior (black) of the inferred scalar advection speed along with volume flow rate *Q* based on the measured cochlear aqueduct cross-sectional area. The fourth column shows the posterior (black) of the Peclet number, Eq. 3. The fifth column shows a posterior-sample pair-plot of the averaged over space diffusivity $\bar{D}$ (y-axis) and the scalar advection speed (x-axis). The bottom row shows results for the 10 cases, all mice and all ears, overlaid on the same figure: posterior-mean diffusivity for each of the 10 cases (first column), the prior and 10 posteriors of the inferred $\sigma _\text {noise}$ (second column), the prior and the 10 posteriors of the inferred scalar advection speed (third column), and 10 posteriors of the inferred Peclet number, Eq. 3 (fourth column). The minimum component-wise effective sample size for all our real-data-based inferences is ∼1100

### Inferred Transport Parameters Indicate that Diffusion Dominates for Small Tracers

The inferred transport parameters include a considerable variety on the profile of the diffusion coefficient, though it dominates or is at the level with advection in most cases (see Fig. 4 for representative and A5 for remaining recordings). The spatial variation is always within 50% of its mean, typically around $\bar{D} \approx 430$µm$^2$/s. The free diffusivity ($D_E \approx 368$µm$^2$/s, in Eq. 1) is within the 68%-credibility interval, which indicates that Taylor-dispersion and oscillatory flow do not contribute much to the evolution of small tracer concentrations in the cochlear aqueduct, or that they are balanced out by reduced diffusion through membranes [12] (Fig. 4 column 1, incl. bottom row). The inferred diffusivity is the same whether advection is permitted or not, as seen in the overlap of gray (including advection) and blue credibility intervals (excluding advection).

For the advection parameter *a*, some recordings gave inferred probability distributions that were indistinguishable from their priors, indicating that the data gave no information on advective contributions to the tracer concentrations (column 3). In other cases, only a little posterior probability remained near 0. However, advection was never more than a few microns per second, corresponding to a Péclet number rarely larger than 1 (column 4).

The inferred noise level was always well within the probable range of the prior, confirming that the noise was represented well in this framework. Similarly, no overt correlation was found in the inferred diffusion and advection estimates, supporting that they are inferred independently (column 5). See Table 1 for a summary of the inferred parameters for all cases.

**Table 1:** Parameter estimates for all recordings (mean of posterior distribution)

Mouse, side	$\bar{D}$ (µm$^2$/s)	*a* (µm/s)	Pe	$\sigma _\text {noise}$
mouse 1, l	427	−0.01	0.00	4.0
mouse 1, r	434	0.64	0.48	2.8
mouse 2, l	438	0.01	0.00	2.9
mouse 2, r	437	−0.42	−0.28	2.5
mouse 3, l	423	0.02	0.01	3.9
mouse 3, r	429	−0.85	−0.69	2.9
mouse 4, l	439	−0.30	−0.22	2.2
mouse 4, r	427	−1.05	−0.89	2.8
mouse 6, l	427	−0.02	−0.02	3.7
mouse 6, r	428	−0.68	−0.49	2.5
mean	430	−0.27	−0.21	3.0
std. dev	5.6	0.5	0.4	0.6

### Approximate Closed-form Solution for Cochlear Aqueduct Transport of Larger Particles with Constant Diffusion

To generalize transport through the cochlear aqueduct to other molecules and animals, we can apply known solutions to the advection–diffusion equation in the case of constant diffusivity (see, e.g., [27]). First, we solve the steady-state of our model Eq. 2$(\partial c(x,t)/\partial t =0)$ with diffusion approximated as constant and dependent on particle size. This approximation holds within 20% error in most of our recordings, see Fig. A4. If we use the position relative to aqueduct length, $s \equiv x/x^\text {max}$, and take the boundaries to have concentrations $c(s=0)=c_0$ and $c(s=1)=c_1$, then the steady-state concentration profile has the form

4 $$\begin{aligned} c(s) = \frac{c_0 e^{\textrm{Pe}} - c_1 + (c_1 - c_0) e^{\textrm{Pe} s}}{e^{\textrm{Pe}} - 1}. \end{aligned}$$

As the Péclet number approaches zero, *c*(*s*) approaches $c(s)=c_0 + (c_1-c_0) x / x^\text {max}$ and we notice that steady-state concentration is independent of transport parameters and therefore useless for inferring them, which instead require dynamic information. The net transport rate can be calculated with knowledge of the aqueduct cross-sectional area *A* as

5 $$\begin{aligned} J = Q \frac{c_0 e^{\textrm{Pe}} - c_1}{e^{\textrm{Pe}} - 1}, \end{aligned}$$

where Q=Aa is the volume flow rate. As the Péclet number approaches zero, the net transport approaches

6 $$\begin{aligned} J = A D (c_0 - c_1) / x^\text {max} \end{aligned}$$

(as can be checked using, e.g., L’Hôpital’s rule). This formula provides an estimate of the transport rate correct within a factor of ten, an order of magnitude estimate.

To estimate the transport rate through cochlear aqueduct including advection for a general particle of interest with weight *w*, researchers can calculate the Péclet with the following formula and plug it into the transport formula Eq. 5.

7 $$\begin{aligned} \textrm{Pe} = \frac{a x^\text {max}}{\bar{D}} \approx a x^\text {max} \frac{6 \pi \eta }{k_B T} \left( \frac{3w}{4\pi \rho }\right) ^{1/3}. \end{aligned}$$

For proteins, the density is typically $\rho \approx 8.1 \times 10^{29}$Da/m$^3$, the viscosity can be taken $\eta \approx 10^{-3}$Pa s, body temperature T≈310K and the Boltzmann constant is $k_B \approx 1.38\times 10^{-23}$J/K. If we take $x^\text {max}=300$µm and advection a=1µm/s, the Péclet number comes out to $\textrm{Pe} \approx 0.1w^{1/3}$, such that a molecular weight of 1kDa corresponds to a Péclet number of 1, while a weight of 1MDa corresponds to a Péclet number of 10.

In sum, researchers can now calculate an estimate of the rate of transport through the cochlear aqueduct in any animal for small solutes of their interest for given assumptions on net flow and molecular weight. Particles as large as viruses are known to traverse the aqueduct, but whether membranes interfere with these transport modes remains unknown [12, 13].

## Discussion

Transport in the cochlear aqueduct of a non-perforated cochlea has been challenging to quantify due to its small size, and the extent to which communication occurs between the cerebrospinal fluid on one side and cochlea fluids on the other side remains unclear. Using live imaging of a small CT tracer injected in the cisterna magna, relatively far from the cochlea, we show here that a simple 1-dimensional advection–diffusion model is sufficient to predict cochlear aqueduct transport of small inert molecules. Though we allowed advection in addition to diffusion, a model with constant free diffusion does nearly as well in predicting small tracer transport.

The inferred distributions of advection mostly overlapped considerably with zero and were rarely great than 1µm/s, meaning that for the small tracer studied, advection barely contributed and the Péclet number was usually less than 1 in magnitude. Since the formula for free diffusion does well in predicting tracer concentrations, we can cautiously extrapolate these results to larger molecules and even particles using the Péclet number, which is then proportional to the (hydrodynamic) radius of the particle. As an order of magnitude estimate, advection would dominate when the Péclet number is at least 10, requiring therefore a molecule ten times bigger by radius than our CT tracer, or a thousand times bigger by volume (and therefore weight). Future experiments with large tracers would be ideal for validation of the results presented here.

The direction of the inferred advection varied between the recordings. This could be due to noise, but could also reflect true flow. Early suggestions that blood is the source of four-fifths of perilymph in guinea pigs [14, 15] align with the classical Starling model of peripheral interstitial fluid flow due to osmosis. The more recent large estimates of flow also considered absorption into blood as a main option [18]. Osmosis has recently been proposed to drive flow through brain tissue with a direction reflecting the relative osmolarity of blood versus tissue at a given time, which could also be the case for the cochlea [33]. Alternatively, slow cochlea blood volume oscillations may drive flows, as has been observed in the brain [34]. We have here assumed constant flow parameters which may correspond to time-averages of very fast dynamics, but risk bias if the dynamics are on the same time-scale as our recordings (5–30 min). If natural flows are bidirectional depending on, e.g., circadian rhythm or the impact of feeding on osmosis, then flow estimates averaged across animal experiments obscure this reality, and it is therefore important to consider the full distribution of estimated transport parameters both within and between animals.

Other approaches to estimating advection through the cochlear aqueduct have reported values ranging from 0.05 [17] to 30nl/min [18] in guinea pigs (our estimates for mice are −0.5 to 0.5nl/min, see Fig. 4). In humans, flow rates have been estimated within the scala tympani up to 100nl/min (at 1Hz oscillation of the basilar membrane, with even higher estimates for lower frequencies) [28]. We can take this as a rough estimate of cochlear aqueduct flow [18]. An order of magnitude scaling relationship can then be outlined. If we take guinea pigs to weigh about 40 times as much as our mice and have a flow rate *Q* of 10nl/min [16] which is a factor ten greater than our maximal estimates for mice, then this suggests that the flow rate goes in proportion to average animal area: With area *A* and weight *W* we have $Q \propto A \propto W^{2/3}$ since $10 \approx 40^{2/3}$. Humans are about 4000 times heavier than our mice, which is the right order of magnitude for this scaling law since $100 \approx 4000^{2/3}$. With constant pressure across the species, the cochlear aqueduct area can then not also scale with the species, and this is confirmed by measurements in rats, guinea pigs, and humans, which all have similar aqueductal cross sections [2, 4, 35]. Across animals from mice to humans, cochlear aqueduct flow rates may therefore scale with the surface area of, e.g., scala tympani and vestibuli.

A methodologically similar approach, including Bayesian inference of advection- and constant diffusion-parameters, has previously been employed to investigate transport of a magnetic resonance tracer through brain tissue [21]. In that study, a tracer of similar size (0.6kDa) was reported to flow with speeds near 0.04µm/s within tissue, corresponding to a Péclet number just under 1 (over the longer distance of the whole brain). The nearly even contribution of advection and diffusion requires the inclusion of both in modeling and data interpretation.

One limitation in our choice of prior is the lack of precise measurements of the viscosity of cerebrospinal fluid η, which impacts the diffusion coefficient of iohexol, which also has not been determined in cerebrospinal fluid. We used the measurements by Bloomfield et al [24] in which 2 of 23 subjects had η>0.9mPa s, which would reduce our prior from $D_E\approx 369$µm/s to $D_E\approx 259$µm/s, in which case our estimates of diffusion may reflect contributions from oscillations. We ran inferences with wider Gaussian priors without affecting our main results. We also tried priors that impose spatial correlation, in particular, modeling the diffusivity random variable to be a discretized Gaussian Markov Random Field (GMRF). Although in some cases GMRF-based inference gives qualitatively similar predictions and posterior mean, the spatial variations in the inferred diffusivity were overly smoothed. We note that our Gaussian prior is discretized on a grid that is three times coarser than the solution grid (“Discretization of the Transport Model” section), which already introduces a degree of regularization and smoothing in the inferred solution. Additional smoothing from a GMRF prior may therefore obscure abrupt changes in diffusivity associated with thin membrane or tissue mesh structures in the aqueduct. Thus, we find no advantage in using GMRF priors in this setting. Our experience shows that when converged, Metropolis-Hastings (MH) samplers give comparable results to the NUTS sampler. However, the MH sampler can suffer from extremely slow convergence, and therefore, we relied on the NUTS sampler. We provide code for complete replication of the presented results, which can also be used to modify Bayesian modeling and sampling choices.

We were here limited to studying male mice anesthetized with ketamine/xylazine, which may have effects on cochlear aqueduct transport. Future modeling work may take advantage of the 3-dimensional CT imaging data to account for the initial funnel of the cochlear aqueduct, as well as to investigate transport within the scala tympani, where tracer is also detectable. A 3-dimensional model might enable identification of membranes and other structures. We used only males, so future studies should image females too. We also note that the injection pump, in principle, could modify cerebrospinal fluid flow patterns around the cochlear aqueduct, but this is unlikely since the pump was off during the image acquisitions and other studies show that the injection pump does not modify bulk flow patterns [36].

Gene therapy to rescue hearing is evolving with clinical trials ongoing [6, 7]. Most studies in mice and humans use direct injection into the inner ear, which have the risk of damaging the inner ear structures [6, 7]. The aqueduct patency in humans is debated, but three studies report more than 90% of the specimens either fully patent or partly patent [1, 2, 37]. Using measures of the displacement of the tympanic membrane to measure changes in intracranial pressure during postural changes, the aqueduct was found to be functionally patent in more than 70% of adults [38, 39]. Totten et al. found that when injecting MR contrast agent into the cerebrospinal fluid in 15 human patients, the contrast agent was observed in the cochlea in all of the patients and first reaching the basal turn, indicating it entered through the cochlear aqueduct [40]. Viral expression in the opposite ear has been reported following injections of AAVs directly into cochlea [41]. After diffusion from the cochlear aqueduct, the convective flows in the subarachnoid space can likely transport the viruses throughout this space (but this is outside the domain of the present model).

## Conclusion

In CT imaging of a small tracer in the intact cochlear aqueduct of anesthetized mice, free diffusion is sufficient to predict the evolution of the tracer signal. Advection in either direction may contribute, though always slower than 2 µm/s. meaning that transport of small molecules in the mouse cochlear aqueduct is likely governed by free diffusion.

## Methods

### Animals and Imaging

#### Animals

6 C57BL/6 8-weeks old male mice (Janvier Labs, weight 24–26 g) were used for the experiment. The mice were housed in groups of 3 mice per cage with controlled temperature and humidity on a 12 h/12 h light/dark cycle and were fed ad libitum. The experiments were done during the light phase. The experiment was approved by the Animal Experiments Council under the Danish Ministry of Environment and Food (license number: 2015-15-0201-00535 and 2020-15-0201-00480). The procedures were performed in accordance with the European directive 2010/63/EU, with due care to minimize the number of animals included in the study.

#### Cisterna Magna Cannulation

The protocol used in this study to inject tracer into the cisterna magna (CM) is an adaptation from the chronic cannula implantation procedure previously described [42]. Briefly, the mice were anesthetized with a mixture of Ketamine/Xylazine (100 mg/kg, 10 mg/kg, respectively) and after depth of anesthesia was confirmed by the cessation of reflexes, the incision area was properly shaved and sterilized with the aid of ethanol 70% swabs (Vitrex Medical A/S) followed by iodine solution (povidone iodine 7,5 procent Henry Schein). Lidocaine (0.05 ml, 0.2 mg/ml) was applied in the incision site, and Buprenorphine (0.05 mg/kg) injected subcutaneously for postsurgical analgesia. A skin incision was made over the occipital crest, and the neck muscles were separated to reveal the atlanto-occipital membrane. A 30 G needle (SOPIRA®Carpule 30 G 0.3 12 × 12 mm, Kulzer) attached to a PE 10 tube filled with CT tracer Iohexol (Omnipaque 350 mg I/ml, GE Healthcare) was inserted into the cisterna magna. The PE tube was previously connected to a syringe (Hamilton syringe GASTIGHT, 1700 series, 1710TLL, 100-µl volume, PTFE Luer lock), which was placed into a Syringe pump (LEGATO 130 Syringe pump, KD Scientific). A total volume of 10 μl was injected at a rate of 1 μl/min.

#### Computed Tomography (CT) Imaging

Immediately after the CM cannulation procedure, the mice were moved to the CT scanner. A custom-made head bar was mounted on top of the head during the CM cannulation. The head bar was designed to minimize undesired movements during the image acquisition[43]. Body temperature was maintained at 37 ± 0.5°C with a thermostatically controlled heating pad and the respiratory rate observed by a respiration monitoring system (BioVet™, m2m Imaging Corp). All CT scans were performed using a Vector4uCT system (MILabs) with isometric resolution of 20 μm (50 kV, 0.18 mAs, 0.25 deg rotation/scan, 0.0 pitch, Al500 filter, FOV 54 × 54 × 140 mm3, matrix 2700 × 2700 × 7000). The caudal portion of the mice head containing the inner ears was scanned; every single scan took 4 min and 56 s. The time series CT scanning protocol comprised one baseline scan followed by an intracisternal infusion of iohexol. After the tracer injection, 6 continuous scans were acquired over 30 min. Acquired images were processed as described below. One animal was excluded due to no tracer influx observed, probably due to the disconnection of the cannula when the animal was moved to the scanner.

#### Image Processing

CT time series data were motion-corrected using Advanced Normalization Tools (ANTs ver.2.1.0) [44, 45]. After motion correction of the CT data, time-intensity data was extracted using ITK-SNAP (ver. 3.8.0, www.itksnap.org)[46] with the regions of interest previously described in Mathiesen et al. 2023 [4]. Distances between the ROI were calculated using their spatial coordinates in ITK-SNAP.

### Modeling the Bayesian Inverse Problem

#### The Transport Model

We model the time- and space-varying tracer concentration *c*(*x*, *t*) measured by CT according to the advection–diffusion equation

8a $$\begin{aligned} 0&= \frac{\partial c(x,t)}{\partial t} - \frac{\partial }{\partial x}\left( D(x) \frac{\partial c(x,t)}{\partial x} \right) +a\frac{\partial c(x,t)}{\partial x},\nonumber \\&x\in [0,x^\text {max}], \; t \in [0, t^\textrm{max}], \end{aligned}$$

8b $$\begin{aligned} c(0,t)&= c_0(t), \quad t \in [0, t^\textrm{max}]\end{aligned}$$

8c $$\begin{aligned} c(x^\text {max},t)&= c_1(t), \quad t \in [0, t^\textrm{max}]\end{aligned}$$

8d $$\begin{aligned} c(x,0)&= g(x), \quad x\in [0,x^\text {max}]. \end{aligned}$$

Here $t^\text {max}$ is the duration of the CT recording. Equations 8b and 8c are the left and right Dirichlet boundary conditions, respectively. We obtain the value of the signals $c_0(t)$ at x=0 and $c_1(t)$ at $x=x^\text {max}$ from the CT data. Equation 8d is the initial condition where the initial concentration *g*(*x*) is also obtained from the CT data at time t=0. The length of the domain $x^\text {max}$ differs from one mouse/ear to another and ranges from ∼288 µm to ∼356 µm and $t^\text {max}=30$ min. for all cases.

#### Discretization of the Transport Model

We discretize Eq. 8 in time using backward Euler discretization. We choose relatively small time steps for the first five time steps to accurately capture the rapid tracer concentration change as it flows into the domain, steps of size ∼25 s, and take larger steps thereafter, ∼2 min. We use centered-difference discretization for the diffusion operator $\frac{\partial }{\partial x}\left( D(x) \frac{\partial }{\partial x} \;\cdot \right)$ and the advection operator $a\frac{\partial \;\cdot }{\partial x}$. We specify the finite difference grid representing the interval $[0, x^\text {max}]$ to have ∼60 equidistant nodes.

We denote by $\boldsymbol{c}$ the discretized concentration in time and space and by $\boldsymbol{D}$ the discretized diffusivity in space. Note that to reduce the number of unknowns, we discretize $\boldsymbol{D}$ on a coarser grid (of 21 nodes) and interpolate it on the finer grid when solving Eq. 8.

#### The Forward Map

Here, we provide the details of defining the forward map, which maps the unknown parameters to be inferred to the observable quantities. Evaluating the forward map involves solving the discretized version of Eq. 8 for the concentration $\boldsymbol{c}$ given specified values of the coefficients $\boldsymbol{D}$ and *a*, and the initial and boundary conditions. We denote by $\boldsymbol{S}$ the solution operator (in the discretized setting) which is a map that, for given coefficients, gives the concentration solution, i.e., $\boldsymbol{c}=\boldsymbol{S}(\boldsymbol{D}, a)$. To ensure the positivity of the inferred $\boldsymbol{D}$, we parameterize it by a vector $\boldsymbol{d}$ through the parameterization $\boldsymbol{G}_D({\boldsymbol{d}}):= \boldsymbol{D}={\boldsymbol{d}}^2$, where $.^2$ is the element-wise square operation.

Lastly, we introduce the observation operator $\boldsymbol{O}$ that maps the solution $\boldsymbol{c}$ to the observed quantities, $\boldsymbol{y}$, i.e., $\boldsymbol{y} = \boldsymbol{O}(\boldsymbol{c})$. One option is to choose $\boldsymbol{O}$ to be the operator that extracts the tracer concentration at the CT acquisition times and specified ROI locations. However, in our case, $\boldsymbol{O}$ additionally computes the spatial gradient for these extracted values, which we approximate using the first-order finite difference method; hence, $\boldsymbol{y}$ here is the extracted signal gradient. Our choice is justified on the observation that using a concentration gradient in the Bayesian inference instead of using the concentration signal directly led to more accurate concentration prediction and aligns with how the gradient enters Eq. 8. While $\boldsymbol{y}$ denotes the observable quantity from the numerical solution $\boldsymbol{c}$, we use the symbol $\boldsymbol{y}^\text {obs}$ to denote the actual observed (and processed) data, i.e., the finite-difference spatial-gradient approximation of the measured CT signal.

The forward map, denoted by $\boldsymbol{A}$, is then defined as the composition of the maps defined above as follows:

9 $$\begin{aligned} \boldsymbol{A}(\boldsymbol{d}, a) := (\boldsymbol{O} \circ \boldsymbol{S}) (\boldsymbol{G}_D(\boldsymbol{d}), a) = \boldsymbol{y} \end{aligned}$$

#### Bayesian Inverse Problem

In our study, we are interested in solving the inverse problem of inferring the unknown parameters $\boldsymbol{d}$ and *a* using the observed, often noisy, data $\boldsymbol{y}^\text {obs}$. In this case, the noise stems from the CT measurement process along with the ROI averaging. We assume that the noise in the signal is additive, and independent and identically distributed (i.i.d.) Gaussian noise. The additive noise assumption is justified by the standard deviation of the regions of interest being virtually independent of the mean signal magnitude (see Fig. A6). We denote by $\sigma _\text {noise}$ the standard deviation of the noise.

Thus, in the Bayesian setting, we consider $\boldsymbol{y}$ a vector-valued random variable with i.i.d Gaussian components centered at the computed concentration gradient $\boldsymbol{A}(\boldsymbol{d}, a)$, and with a standard deviation $\sigma _\text {noise}$. We parameterize $\sigma _\text {noise}$ by the precision, which we denote as δ, i.e., $\sigma _\text {noise}= \delta ^{-\frac{1}{2}}$. Because we do not know the noise level exactly, we add a prior distribution for δ to enable the Bayesian framework to estimate this ‘hyperparameter’ δ in addition to the unknown parameters $\boldsymbol{d}$ and *a*. We choose a Gamma distribution to model δ since it enforces positive inferred values of δ, can be tuned to be wide enough so that it enables inferring a wide range of noise levels, and can be sampled directly utilizing conjugacy relations (in the case of additive Gaussian noise). Lastly, we model *a* and $\boldsymbol{d}$ as a scalar and a vector-valued random variables, respectively, following a Gaussian distribution with specified mean and variance. Unless specified otherwise, from this point forward, we denote by $\boldsymbol{d}$, *a*, and $\boldsymbol{y}$ the diffusivity, advection, and data random variables (as opposed to deterministic values). The complete Bayesian model is thus written as

10a $$\begin{aligned} a&\sim \textrm{Gaussian}(0, \sigma _a^2)\end{aligned}$$

10b $$\begin{aligned} \boldsymbol{d}&\sim \textrm{Gaussian}(\bar{\boldsymbol{d}}, \sigma _d^2\boldsymbol{I})\end{aligned}$$

10c $$\begin{aligned} \delta&\sim \textrm{Gamma}(\alpha , \lambda )\end{aligned}$$

10d $$\begin{aligned} \boldsymbol{y}|\boldsymbol{d}, a, \delta&\sim \textrm{Gaussian}(\boldsymbol{A}(\boldsymbol{d}, a), \delta ^{-1} \boldsymbol{I}), \end{aligned}$$

where we choose the values of $\sigma _a$, $\bar{\boldsymbol{d}}$, and $\sigma _d$ to be 0.8 µm/s., 20 µm$/\sqrt{\text {s}}$, and 5 µm$/\sqrt{\text {s}}$, respectively. We specify α=0.9 and λ=0.5 for the control experiments and α=1.2 and λ=5 for the real-data experiments, respectively.

### Sampling the Posterior

The posterior distribution of the unknown parameters and the hyperparameter of the Bayesian model in Eq. 10 is given by

11 $$\begin{aligned} p(\boldsymbol{d}, a, \delta | \boldsymbol{y}=\boldsymbol{y}^\text {obs}) \propto L(\boldsymbol{d}, a, \delta | \boldsymbol{y}=\boldsymbol{y}^\text {obs}) p(\boldsymbol{d})p(a)p(\delta ), \end{aligned}$$

where $\boldsymbol{y}^\text {obs}$, the data, is a particular realization of the data random variable $\boldsymbol{y}$. $p(\boldsymbol{d}, a, \delta | \boldsymbol{y}=\boldsymbol{y}^\text {obs})$ is the posterior distribution of $\boldsymbol{d}$, *a*, and δ given the observed data $\boldsymbol{y}^\text {obs}$. $p(\boldsymbol{d})$, *p*(*a*), and p(δ) are the prior probability density functions (PDFs) of the random variables $\boldsymbol{d}$, *a*, and δ, respectively. We denote by $p(\boldsymbol{y}|\boldsymbol{d}, a, \delta )$ the PDF of the data $\boldsymbol{y}$. Fixing the value of $\boldsymbol{y} = \boldsymbol{y}^\text {obs}$ and allowing this PDF to change over the conditioning variables $\boldsymbol{d}, a, \delta$ gives what is called the likelihood function $L(\boldsymbol{d}, a, \delta | \boldsymbol{y}=\boldsymbol{y}^\text {obs}):= p(\boldsymbol{y}=\boldsymbol{y}^\text {obs}|\boldsymbol{d}, a, \delta )$.

We use the NUTS algorithm [47] to sample the parameters $\boldsymbol{d}$, *a*, and we sample δ directly by utilizing conjugacy in a hybrid Gibbs framework. Since NUTS sampling requires the gradient of the log posterior with respect to the unknown parameters, we use the finite difference method to approximate the gradient with respect to $\boldsymbol{d}$ and *a*. For each of the control and real-data experiments, we generate a chain of ∼2000 samples. We set the NUTS step size to a fixed step, 0.1, and set the maximum tree depth to 10. In all the control and real-data cases we present, we obtain a high component-wise effective sample size, above ∼1000, indicating satisfactory convergence.

#### Implementation Aspects

We implement solving this Bayesian problem using the software tool Computational Uncertainty Quantification for Inverse Problems in Python (CUQIpy)^1^ [30, 31]. CUQIpy is a software tool for modeling and solving Bayesian inverse problems as well as processing and visualizing the obtained solution. It provides flexible Bayesian hierarchical modeling capabilities, including a wide range of prior choices and flexible noise modeling. It also provides classical, advanced, and Gibbs-based sampling methods. It supports various types of forward models, e.g., linear, nonlinear, and models based on partial differential equations.

## Supplementary Information

Below is the link to the electronic supplementary material.

Supplementary file 1 (pdf 654 KB)

## Data Availability

To allow full reproducibility of our results in this paper, we provide all code open source (On the version control repository https://github.com/CUQI-DTU/Paper-Cochlear and on the archiving repository https://doi.org/10.5281/zenodo.15796673 (see reference [32]).).

## Footnotes

1 https://cuqi-dtu.github.io/CUQIpy.

## Declarations

### Use of AI

Artificial intelligence was not used in making this manuscript.
