# Supplementary material for: Cochlear Aqueduct Advection and Diffusion Inferred from Computed Tomography Imaging with a Bayesian Approach
Source: J Assoc Res Otolaryngol. 2026 Apr 13;27(4):555–67. doi: 10.1007/s10162-026-01047-x (PMC13504145; doi:10.1007/s10162-026-01047-x)
Supplement: Supplementary file 1 [file 10162_2026_1047_MOESM1_ESM.pdf]

# Supplementary Information for: Cochlear aqueduct advection and diffusion inferred from computed tomography imaging with a Bayesian approach

Amal M A Alghamdi[1], Barbara K Mathiesen[2], Leo Miyakoshi[2], Maiken Nedergaard[2], Jakob S Jørgensen[1], Peter A R Bork[2,3*]

[1]Department of Applied Mathematics and Computer Science, Technical University of Denmark, Richard Petersens Plads, Building 324, Kongens Lyngby, 2800, Denmark.
[2]Center for Translational Neuromedicine, University of Copenhagen, Blegdamsvej 3B, Copenhagen, 2200, Denmark.
[3]Department of Physics, Technical University of Denmark, Building 307, Kongens Lyngby, 2800, Denmark.

*Corresponding author(s). E-mail(s): peter.bork@sund.ku.dk;
Contributing authors: amaal@dtu.dk; barbara.mathiesen@gmail.com; lmiyakoshi@sund.ku.dk; nedergaard@sund.ku.dk; jakj@dtu.dk;

## Figures A1-A6

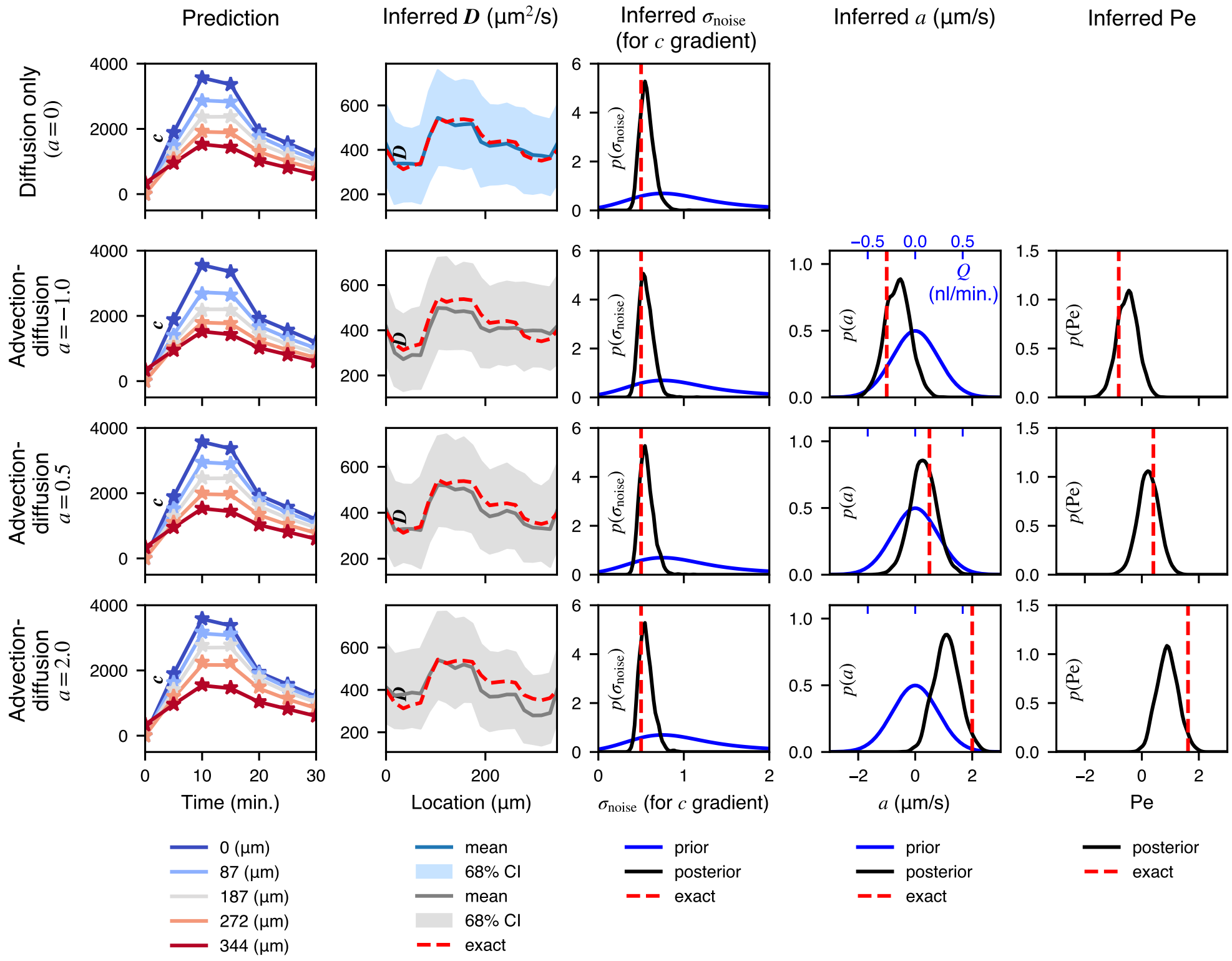


**Fig. A1**: The Bayesian inference setup correctly identifies spatially varying diffusion, advection, and noise in synthetic data for a diffusivity profile of relatively high diffusivity in the middle. Each row represents a different set up of the inference problem: diffusion only (first row), advection of -1 μm/s. (second row), advection of 0.5 μm/s. (third row), and advection of 2 μm/s. (forth row). First column shows the predicted data (solid lines) and the exact data (marked as stars). The second column shows the 68% credibility interval plot of the inferred varying in space diffusivity (blue for the diffusion only case and gray for the advection-diffusion cases), the exact diffusivity profile (red) and the posterior-mean diffusivity profile (blue for the diffusion only case and gray for the advection-diffusion cases). The third column shows the prior (blue) and posterior (black) and exact value (red dashed line) of the noise standard deviation. The fourth column shows the prior (blue) and posterior (black) and exact value (red dashed line) of the scalar advection speed along with volume flow rate $Q$ (based on measured cochlear aqueduct cross-section). The fifth column shows the posterior (black) and exact value (red dashed line) of the Peclet number, equation 3. The minimum component-wise effective sample size for all inferences is ∼1000.

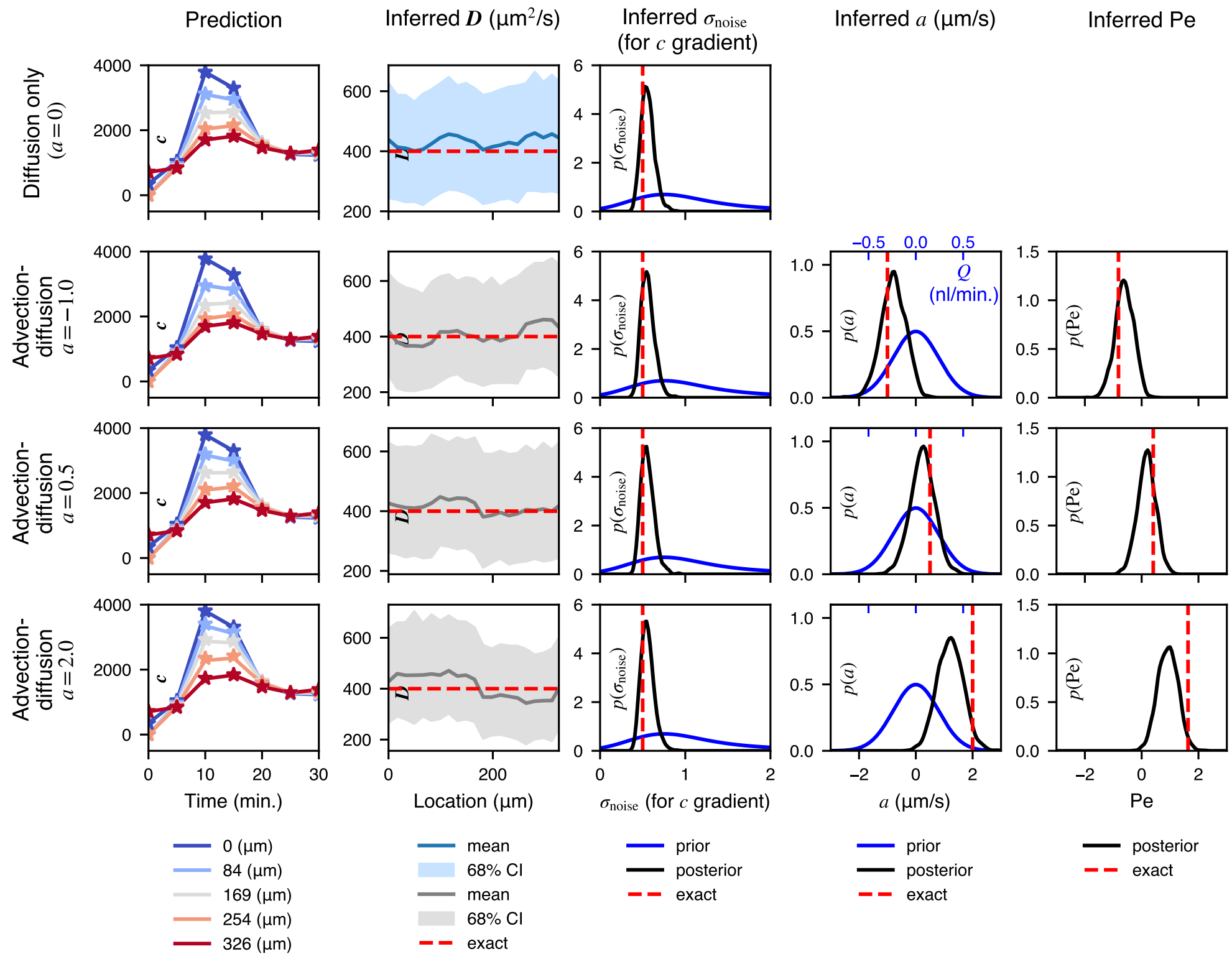


**Fig. A2**: The Bayesian inference setup correctly (qualitatively) identifies constant diffusion, advection, and noise in synthetic data. Each row represents a different set up of the inference problem: diffusion only (first row), advection of -1 μm/s. (second row), advection of 0.5 μm/s. (third row), and advection of 2 μm/s. (forth row). First column shows the predicted data (solid lines) and the exact data (marked as stars). The second column shows the 68% credibility interval plot of the inferred varying in space diffusivity (blue for the diffusion only case and gray for the advection-diffusion cases), the exact diffusivity profile (red) and the posterior-mean diffusivity profile (blue for the diffusion only case and gray for the advection-diffusion cases). The third column shows the prior (blue) and posterior (black) and exact value (red dashed line) of the noise standard deviation. The fourth column shows the prior (blue) and posterior (black) and exact value (red dashed line) of the scalar advection speed along with volume flow rate $Q$ (based on measured cochlear aqueduct cross-section). The fifth column shows the prior (blue) and posterior (black) and exact value (red dashed line) of the Peclet number, equation 3. The minimum component-wise effective sample size for all inferences is $\sim$1200.

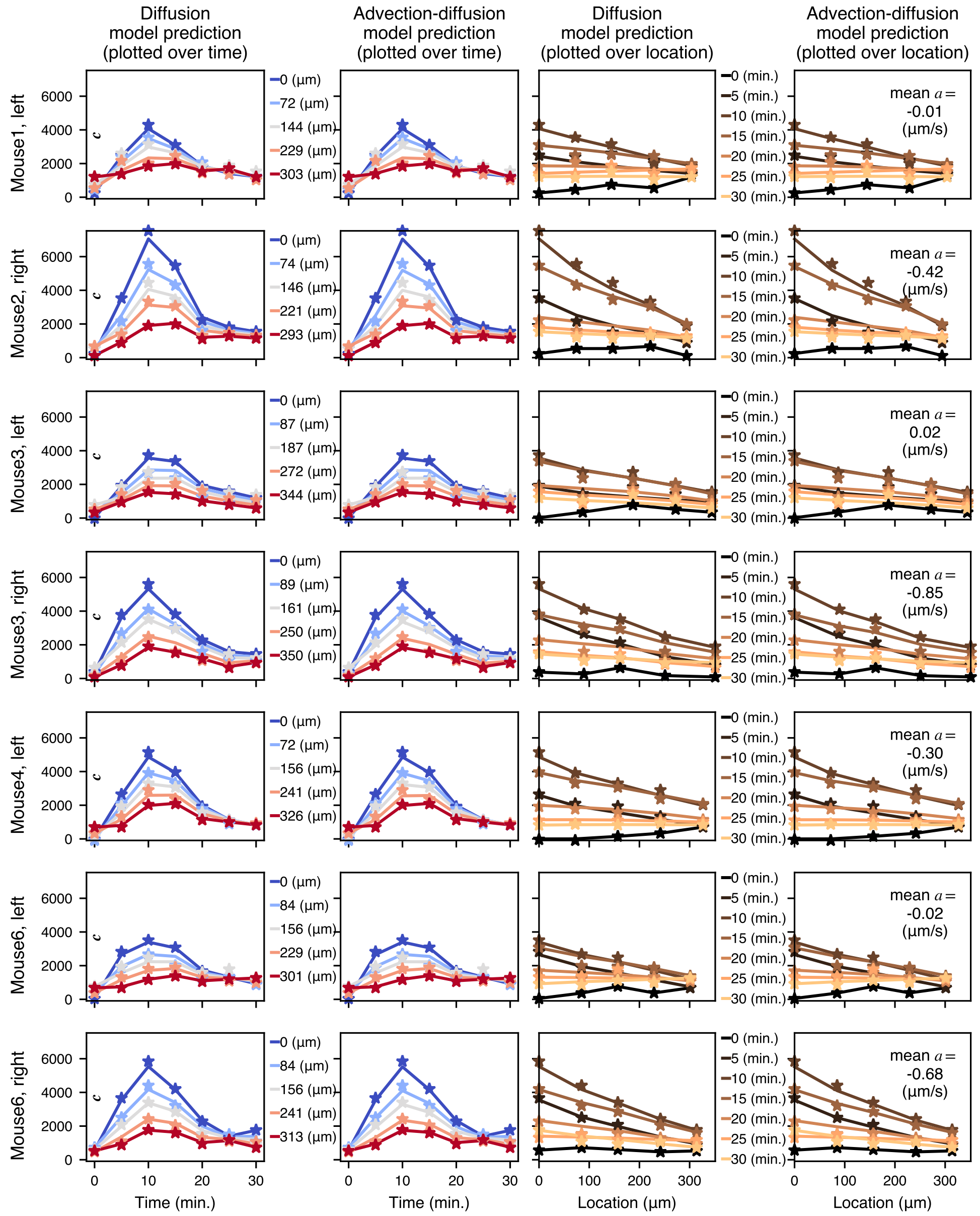


**Fig. A3**: The inferred evolution of tracer concentration in cochlear aqueduct based on real data with and without advection assumption aligns well with the data. This figure shows the remaining cases that were not highlighted in Figure 3. Each row shows prediction for a particular real-data case as labeled. The first column shows our diffusion-only model prediction while the second column shows our advection-diffusion model prediction. Prediction is shown as solid lines and the CT data are marked by stars. The third and fourth columns show that same data in the first and second columns, respectively, but with different visualization: plotting concentrations at specific times over location instead of plotting concentrations at specific locations over time.

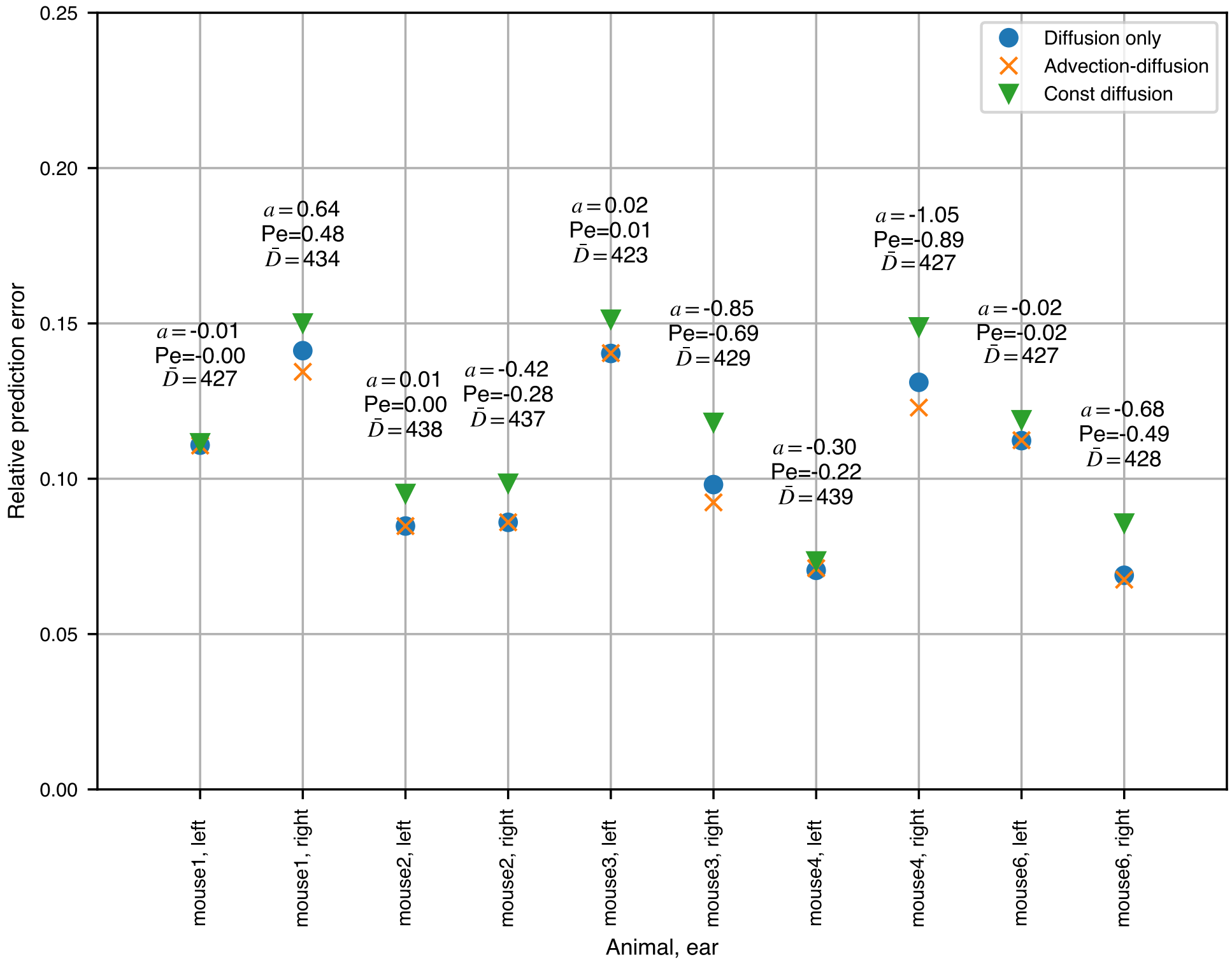


**Fig. A4**: Prediction error for the diffusion-only model (blue circles), the advection-diffusion model (orange $\times$ marks) and the constant-diffusion model (green triangles) for all the 10 cases. The relative prediction error is computed as $||\boldsymbol{c}^{\text{obs}} - \boldsymbol{c}^{\text{model}}||/||\boldsymbol{c}^{\text{obs}}||$, where $\boldsymbol{c}^{\text{obs}}$ is the observed concentrations at the specified times and ROI, and $\boldsymbol{c}^{\text{model}}$ is the corresponding model-predicted concentrations. For each case, the note above the datapoints give the posterior sample mean of the velocity $a$, the corresponding Peclet number Pe, and $\bar{D}$ computed from the diffusion-only model inference.

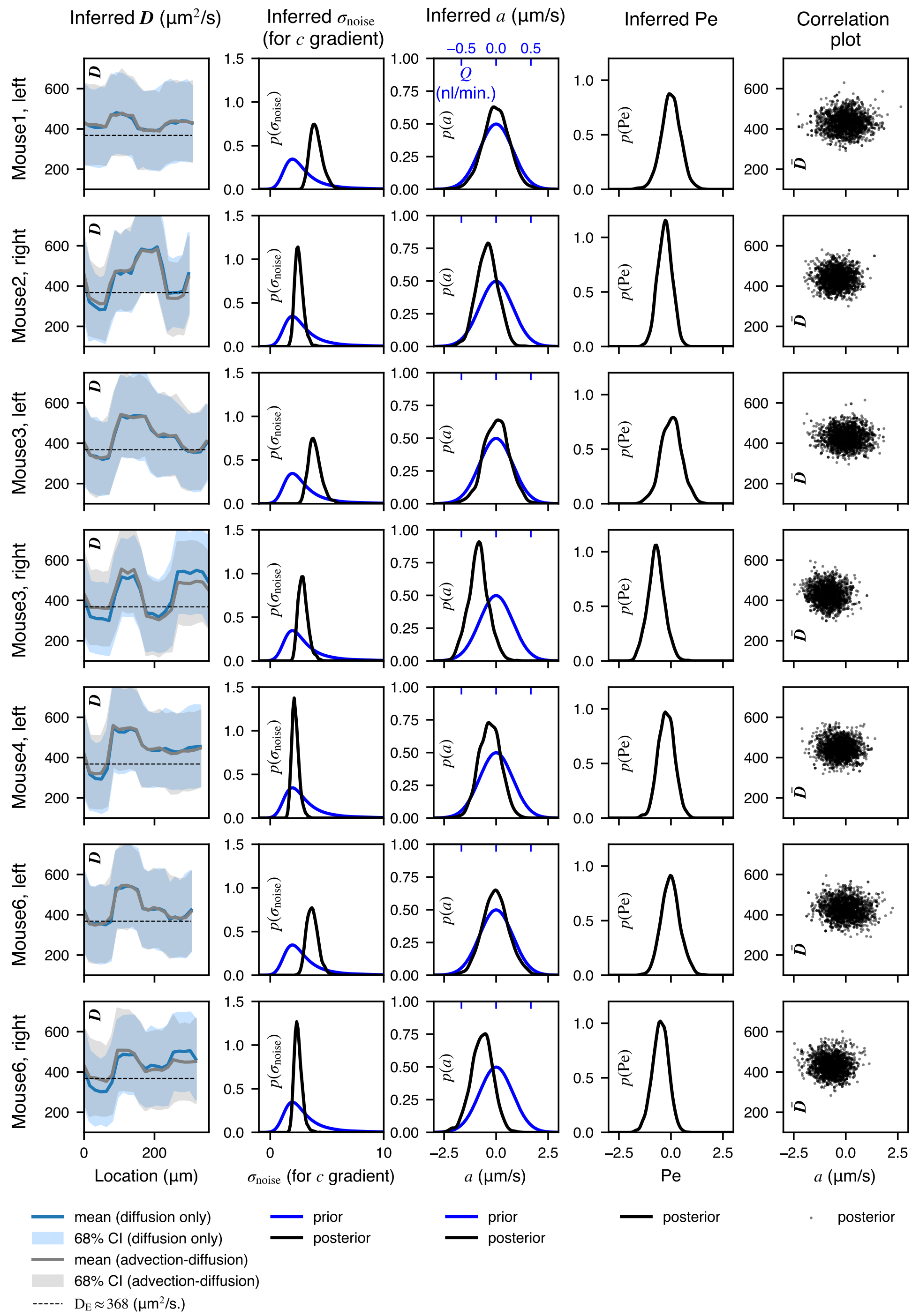


**Fig. A5**: Bayesian parameter inference of spatially varying diffusion near the free diffusivity and small advection levels for the 6 remaining cases that were not highlighted in Figure 4. Each row corresponds to one case, as labeled. In each of those rows, the first column shows the 68% credibility interval plot of the inferred varying in space diffusivity (blue for the diffusion-only inference case and gray for the advection-diffusion inference case), the posterior-mean diffusivity profile (blue for the diffusion only case and gray for the advection-diffusion case) along with $D_{\mathrm{E}}$ value (gray dashed line). The second column shows the prior (blue) and posterior (black) of the inferred noise standard deviation $\sigma_{\mathrm{noise}}$. For simplicity, we only show $\sigma_{\mathrm{noise}}$ posterior for the advection-diffusion case. The corresponding posterior for the diffusion-only case is almost identical. The third column shows the prior (blue) and posterior (black) of the inferred scalar advection speed along with volume flow rate $Q$ based on measured cochlear aqueduct cross-sectional area. The fourth column shows the posterior (black) of the Peclet number, equation 3. The fifth column shows posterior-sample pair-plot of the averaged over space diffusivity $\bar{D}$ (y-access) and the scalar advection speed (x-axis). The minimum component-wise effective sample size for all our real-data-based inferences is $\sim$1000.

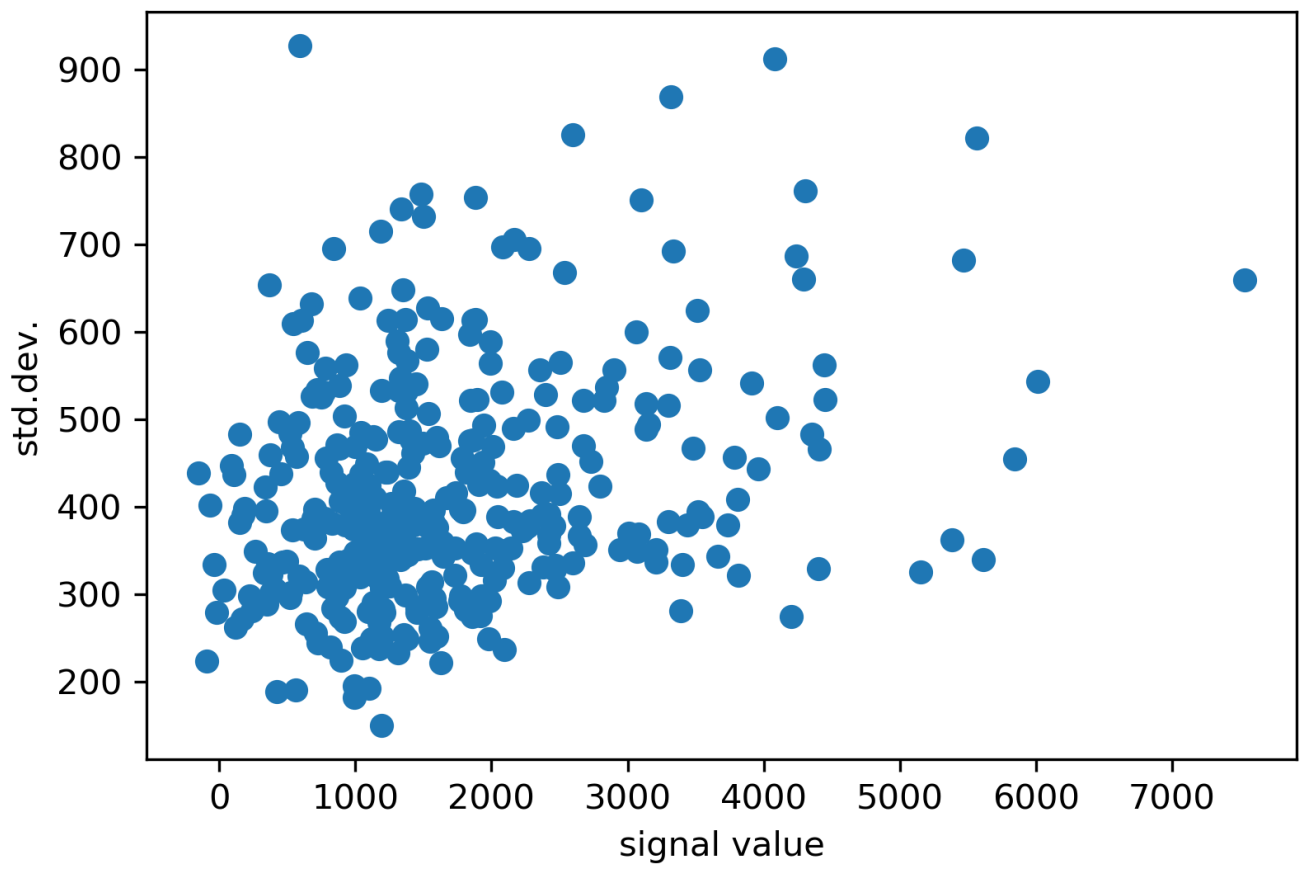


**Fig. A6**: The mean signal value and corresponding standard deviation of each region of interest plotted against each other show no overt correlation.
